# Context Matters: NOTCH Signatures and Pathway in Cancer Progression and Metastasis

**DOI:** 10.3390/cells10010094

**Published:** 2021-01-07

**Authors:** Julia O. Misiorek, Alicja Przybyszewska-Podstawka, Joanna Kałafut, Beata Paziewska, Katarzyna Rolle, Adolfo Rivero-Müller, Matthias Nees

**Affiliations:** 1 Department of Molecular Neurooncology, Institute of Bioorganic Chemistry Polish Academy of Sciences, ul. Noskowskiego 12/14, 61-704 Poznan, Poland; jmisiorek@man.poznan.pl (J.O.M.); kbug@ibch.poznan.pl (K.R.); 2 Department of Biochemistry and Molecular Biology, Medical University in Lublin, ul. Chodzki 1, 20-093 Lublin, Poland; alicja.przybyszewska@umlub.pl (A.P.-P.); joanna.kalafut@umlub.pl (J.K.); beata.paziewska@umlub.pl (B.P.); adolfo.rivero-muller@umlub.pl (A.R.-M.); 3 Institute of Biomedicine and Western Cancer Centre FICAN West, University of Turku, 20101 Turku, Finland

**Keywords:** Notch signaling pathway, tumor progression, oncogenic mutations, tumor suppressor gene, gain and loss of function mutations (GOF and LOF), epithelial-mesenchymal transition (EMT), angiogenesis, metastasis, tumor microenvironment (TME), personalized cancer medicine

## Abstract

The Notch signaling pathway is a critical player in embryogenesis but also plays various roles in tumorigenesis, with both tumor suppressor and oncogenic activities. Mutations, deletions, amplifications, or over-expression of Notch receptors, ligands, and a growing list of downstream Notch-activated genes have by now been described for most human cancer types. Yet, it often remains unclear what may be the functional impact of these changes for tumor biology, initiation, and progression, for cancer therapy, and for personalized medicine. Emerging data indicate that Notch signaling can also contribute to increased aggressive properties such as invasion, tumor heterogeneity, angiogenesis, or tumor cell dormancy within solid cancer tissues; especially in epithelial cancers, which are in the center of this review. Notch further supports the “stemness” of cancer cells and helps define the stem cell niche for their long-term survival, by integrating the interaction between cancer cells and the cells of the tumor microenvironment (TME). The complexity of Notch crosstalk with other signaling pathways and its roles in cell fate and trans-differentiation processes such as epithelial-to-mesenchymal transition (EMT) point to this pathway as a decisive player that may tip the balance between tumor suppression and promotion, differentiation and invasion. Here we not only review the literature, but also explore genomic databases with a specific focus on Notch signatures, and how they relate to different stages in tumor development. Altered Notch signaling hereby plays a key role for tumor cell survival and coping with a broad spectrum of vital issues, contributing to failed therapies, poor patient outcome, and loss of lives.

## 1. Introduction

As tumors grow, they often invade and damage surrounding healthy tissues. As both the result and driver of increasing tumor heterogeneity, altered environmental signaling from the tumor microenvironment (TME), such as stromal cancer-associated fibroblasts (CAFs) and the extracellular matrix (ECM), some tumor cells may acquire an increasingly motile phenotype. Such cells may leave the primary tumor site, penetrate the TME, ECM, and blood and lymph vessels, persist in the blood circulation, and eventually gain the potential to disseminate throughout the entire body. Tumor progression represents a stochastic genetic process, followed by the selection of cells with the most beneficial phenotypic growth advantages. This requires integrating various growth advantages at multiple mechanistic levels that, when combined, support tumor cell survival, neo-angiogenesis, or evasion of the immune system in both the blood stream and the metastatic sites. Successful metastasis must be followed by adaptation to the new and selective conditions found in the host environment, including re-connection to blood vessels for the metastatic lesions to grow. Additional aspects include tumor dormancy (survival of small numbers of tumor cells or cancer stem cells (CSCs) during and after therapy) and increasing “stemness” of tumor cells, thus supporting the self-renewal potential and positively selective advantage of advanced cancer cell populations. This ability to form colonies at new sites represents the primary cause of death in cancer patients and is very prominent in carcinomas, which represent 90% of all solid tumors, and are derived from epithelial cell types. Advanced epithelial cancer cells often show a high level of “cellular plasticity” and can actively alter phenotypic features such as their cytoskeleton, the mode and type of cell motility, or adjust and even hijack normal, developmental differentiation patterns to address their needs in the new environment. For example, epithelial tumor cells may gain mesenchymal properties, acquiring more motile and dynamic traits, which are characteristic for cell types like smooth muscle cells or fibroblasts, or many blood cells. One such mechanism is now well characterized, but far from completely understood: EMT, or epithelial-to-mesenchymal transition. EMT provides strong dynamic, motile, or invasive properties to epithelial cells that tend to depend and thrive on strong cell–cell contacts and adhere strictly to epithelial differentiation programs, until this balance is tipped. Additionally, the type of invasion and motility observed can differ vastly with the environment and conditions. This does not always correspond to our understanding of EMT, and likely represents a highly dynamic and reversible adaptation to growth-supporting niches within the TME. Thus, it is of special interest to understand the complex and interconnected mechanisms behind shape-shifting tumor phenotypes and the many mechanisms of mimicry, engaged by cancer cells. This includes pathways that support tumor cell survival and motility, protect from cell death (AAA or anoikis, apoptosis, and autophagy), and promote overt cancer metastasis.

A central cell communication pathway that steers the type and outcome of cell–cell interactions is Notch signaling. Thus, the aim of this review is to present the most recently gained understanding, related to Notch signaling in the context of these fundamental and extremely dynamic oncogenic processes. To further complicate things, Notch rarely acts alone, and is intricately intertwined with many other signaling pathways and mechanisms.

## 2. Notch Signaling: Principles and Involvement in Physiological and Pathological Processes

The principle of signal transduction in Notch signaling pathway is deceptively simple and requires the entourage of two components: (a) one of the four Notch receptors (Notch1-4) and (b) any one of its five ligands (Jag1-2 or Dll1, -3, and -4). Activation of the canonical Notch signaling then occurs when a Notch receptor-expressing cell (receiving cell) makes physical contact with a Notch ligand-expressing cell (sending cell or also named signaling cell). Conformational changes in Notch receptors, due to mechanical forces exerted by the sending cell, lead to the unmasking of Notch cleavage sites. A series of proteolytic events executed by ADAM metalloprotease and gamma-secretase (γ-secretase) then result in the release of the Notch intracellular domain (NICD) from the membrane, which quickly translocates to the nucleus where it complexes with CSL (suppressor of hairless, also known as RBPJ: recombination signal binding protein for immunoglobulin Kappa J region) and MAML1 (mastermind-like protein 1). This converts the repressing CSL activity into an activator of expression of numerous Notch response genes, including HES1, HES2 and HES5, MYC, CCND1, and VEGF [1].

Under physiological conditions, Notch signaling is essential for vasculature development and angiogenesis during embryogenesis [2] and neo-angiogenesis in wound-healing, tissue repair, or in cancers [3]. Both endothelial cells and smooth muscle cells require sustained Notch signaling for proper vasculature development [4]. In fact, disrupting the balance between DLL and JAG ligands in endothelial cells during vessel formation results in disorganized and poorly perfused vasculature [5]. Expression of key Notch signaling players in adult tissues is more restricted than during development. Nevertheless, cells and tissues forming the adult vasculature remain positive for Notch signaling and express both receptors and ligands. In particular, endothelial and vascular smooth muscle cells express high levels of Notch receptors. This enables them to respond to rapid changes in blood pressure by thickening of the vasculature wall, and is regulated by JAG/Notch signaling [6,7]. Expression of the DLL4 ligand is more restricted and confined to small capillaries and arteries in the adult [8]. Additionally, Notch is involved in the development of the mammary gland and intestinal epithelium, and expressed in stem cells in both adult tissues [9,10]. Activation of Notch receptors in mammary gland stem/progenitor cells can further result in the stimulation of stem cell renewal and subsequent proliferation of progenitor cells [11]. Investigating the true functions of Notch receptors or ligands in the context of living cells and tissues is not straightforward, as mRNA- and protein expression are not directly proportional to the level of pathway activation. Cells with active Notch signaling might even show reduced expression of Notch ligands and/or receptors, depending on protein expression levels within the same cell or neighboring cells (reviewed in [12]). The type and ratio of Notch receptors to ligands; the exact nature of physical contact; the quality, duration, and precise topology of interactions: all of this matters. In addition, different Notch ligands can trigger very different responses, probably due to differential activation rates and variable strength of the corresponding receptors. What matters even more, is whether the ligand(s) and receptors are expressed on the same cell (cis-acting) or in different cells (trans-acting). Finally, each Notch receptor (NOTCH1-4) is by itself able to be activated by any one of the ligands, but downstream signaling may still work differently. NOTCH receptors may even oppose the activity of other sister receptor(s), which may result in very different cellular responses and phenotypes. For example, NOTCH1 is frequently elevated in malignant mesothelioma, while NOTCH2 is reduced. Re-expression of NOTCH2 actively induced cell death in the same cells [13], maybe pointing to conflicting signaling from two different Notch receptors.

**Figure 1:**
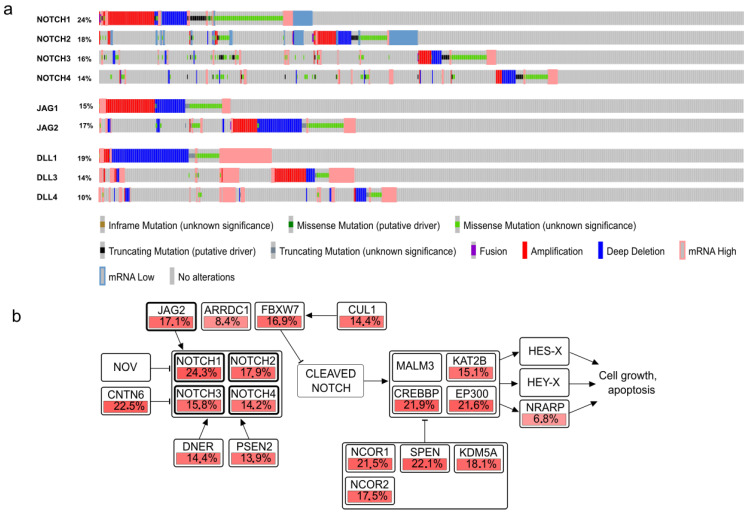
Genetic alterations in Notch pathway genes across human cancer cell lines. (**a**) Mutual exclusivity of genetic events targeting NOTCH receptors and ligands across 1000+ cell lines from the Cancer Cell Line Encyclopedia (CCLE) collection, as displayed in the cBioPortal genome browser. The sorting by mutation type illustrates that genetic mutations affect typically only one of the four NOTCH receptors. Similar mutual exclusivity is observed for the JAG1 and 2, and DLL1, DLL3, and DLL4 ligands. (**b**) Percentage of genetic events (mutations, amplifications, deletions, over-expression) observed in core genes of the Notch pathway, across the 1000+ cell lines of the CCLE collection/BROAD Institute (https://www.cbioportal.org/study?id=ccle_broad_2019). Gene Symbols as identifiers used according to Genome Reference Consortium Human Build 37 (GRCh37).

The analysis of 1000+ cancer cell lines reveals that genetic alterations (mutations, deletions, amplifications, and over-expression) in one NOTCH receptor are usually mutually exclusive to comparable changes in the other three receptors, a pattern likely to be retained from the corresponding original tumors (Figure 1a,b).

Interestingly, gain-of- and loss-of-function (GOF and LOF) mutations are about equally distributed across these cell lines and contribute to the puzzling nature of gain versus loss of Notch functions. Similar mutual exclusivity is observed for some of the ligands. For example, mutation events in JAG1 and JAG2 are almost completely exclusive. Just to add another level of complexity, the Notch signaling pathway also engages in crosstalk with multiple other signaling pathways, maybe most prominently with the WNT, Hedgehog, and hypoxia signaling and the PI3K/AKT/mTOR pathways. Such interactions between signaling molecules also have a plethora of downstream effects (see Section 3.4). The answer for this puzzling ambivalence may relate to the central function of Notch signaling in development. Notch receptors typically transduce signals in response to ligands on neighboring cells, which is critical for lineage-specific differentiation in metazoan tissues, and induction of developmental patterning or segmentation. Only recently, it was demonstrated that conditional activation of Notch in squamous cells activates a context-specific gene expression program through lineage-specific regulatory elements [14]. Any disruption of this differentiation signaling may lead to altered microenvironmental communication in tissues, which may be of benefit for early or later steps in cancer progression, and very much depends on the exact tissue type in which Notch mutations occur. GOF mutations are characteristic for hematopoietic cancers, in which single cells are affected, such as T-cell acute lymphoblastic leukemia (T-ALL) and B-cell chronic lymphocytic leukemia (B-CLL). In solid tissues and cancers, alterations in Notch signaling appear to be bi-directional, and it is the context that matters to which outcome is positively selected for at which stage of cancer initiation or progression, cancer entity, and lineage-specific differentiation switches may be affected. It appears, for example, that early targeted inhibition of the Notch pathway may specifically induce squamous epithelial malignancies. Nevertheless, the high mutation rate of all four NOTCH receptors in a broad spectrum of cancer cell lines across many different tumor entities indicates that these genetic aberrations may preferentially act as driver mutations that promote cell proliferation and survival. Such mutations may also promote progression and survival of cancer cells at later stages of progression in patients.

## 3. The Relevance of NOTCH Mutations, Amplification, Pathways, and Signatures

In 1991, the first human homologue of the *Drosophila* developmental regulator TAN-1 was identified in human T-lymphoblastic leukaemia [15], as the target of chromosomal translocations. At the same time, the first of four human TAN-1 homologues was identified as a critical factor in mammalian embryonal organ and tissue development [16], later also in hematopoietic stem or precursor cells [17]. TAN-1 had soon been renamed into NOTCH1, and two additional human homologues NOTCH2 and NOTCH3 were mapped to their corresponding chromosomal locations [18]. For most of this early time, NOTCH1 and its “new” homologues were mainly considered important differentiation-promoting factors [19] that are highly conserved across species, but strictly confined to developmental processes. Starting from 2000, new data indicated that Notch signaling may also be relevant for the initiation or progression of human tumors, such as small cell lung cancers (SCLCs) [20] and Hodgkin’s and anaplastic large cell lymphoma [21]. The true relevance of oncogenic Notch functions in cancer progression, however, remained elusive (and to some degree, still does today). Nevertheless, these initial findings were already hinting towards the outstanding future relevance of Notch mutations across many neoplasias. Eventually, in the year 2004, the massive impact of recurrent oncogenic point mutations in NOTCH1 were identified in human T-ALL [22], pointing to NOTCH1 as a major proto-oncogene. This seminal finding initiated the mapping of Notch receptors and ligands, and down- and upstream Notch regulatory genes across almost all human cancer entities and subtypes, which continues to this day. Soon, NOTCH1 mutations were also identified in other types of leukemia as well [23]. Despite such compelling evidence, altered NOTCH receptor expression and prominent NOTCH-regulated genes showed mixed and often contradicting effects across different tumor types. Thus, it took much longer to unravel the now classic canonical Notch pathway and Notch-related “gene signatures” in solid human cancers.

Changes in Notch signaling, expression, point mutations, deletions, and amplification/over-expression of Notch-related loci and alleles have since been identified in almost all solid cancers [24,25,26,27]. The question of which exact role Notch signaling may play in tumor initiation versus tumor progression (including drug resistance, dormancy, stemness, relapse, and metastasis) remains unclear and is debated for some tumor entities, maybe with the exception for leukaemia (T-ALL and B-CLL), in which Notch mutations were clearly identified as initiating oncogenic events. This frequent functional uncertainty supports the notion that other signaling pathways linked to Notch may be critical, possibly tipping the balance towards beneficial, selective growth advantages for tumor cells that have either activated or inactivated Notch signaling. This scenario is also supported by mathematical simulations by Vujovic and collaborators [28]. Yet, once the balance is effectively tilted towards the promotion and survival of cancer cells, Notch signaling might increasingly fuel tumorigenesis, either by Notch ON or Notch OFF conditions. Here, we translate this as GOF versus LOF genetic events.

### 3.1. Gain of Function NOTCH Mutations and Their Consequences

Point mutations that result in a gain of function (GOF) in terms of Notch signaling have been most thoroughly investigated in leukemias like T-ALL [22]. Recurrent GOF or oncogenic events in Notch receptors may be relevant also for other hematopoietic cancers. In many other tumor types, oncogenic vs. tumor suppressor functions of Notch may strongly depend on the tissue of origin, the differentiation status, composition of the TME, invasion of immune cells or immune cell evasion, and the genetic background: It is clearly the context that matters most with Notch. For example, genetic GOF events of Notch receptors (amplification or point mutations) that promote Notch activity appear to support the initiation and progression of gliomas (neuronal differentiation, in [29]) and other non-epithelial cancers, such as osteosarcoma (mesenchymal differentiation; in [30]) or SCLCs (neuro-endocrine differentiation, in [31]); although the evidence is controversial: for example, recent articles report both oncogenic and tumor-suppressor functions for NOTCH in glioma [29]. However, also in some carcinomas, or tumors of epithelial origin, such as gastrointestinal (stomach, oesophagus) and colorectal cancers, GOF or oncogenic Notch mutations appear predominant. Activating mutant Notch receptors may further collaborate with frequent p53 mutations [32], thus promoting EMT and enhancing invasive or aggressive phenotypes in various cancer types such as colorectal carcinomas and pancreatic cancer. This will be taken up further below. GOF Notch mutations have been described as promoting EMT and invasive phenotypes in NSCLCs (non-small cell lung cancers) [33], as well as promoting a drug-resistant phenotype, e.g., against gefitinib in lung cancer [34]. Cooperation with recurrent mutations that occur early in tumor progression, maybe most prominently the *p53* tumor suppressor, may further promote Notch-specific effects on EMT and thus enhance aggressive properties [32], as well as promote drug resistance, as demonstrated for cetuximab in HNSCC [35]. These findings in advanced lung cancers are in line with recent discoveries showing that EMT may be dispensable for metastasis, but promotes cell survival and drug resistance [36], possibly with the help of Notch. These findings also prompted us to focus on the role of Notch in EMT-related switches as a major driver towards enhanced tumor aggressiveness and possibly metastasis (Section 4).

One of the well-established consequences of Notch signaling is maintenance of the stem cell pool in various tissues, and the impact of Notch on lineage-specific differentiation switches. Thus, it is not surprising to identify persistent Notch activation and GOF mutations as a means to promote and maintain the stem-cell character or “stemness” of advanced cancer cells, for example in oral cancers [37]. Although a subject of intense debate, these and many other observations point to the possibility that NOTCH1 may act as a tumor suppressor during the initiation of squamous carcinomas [38], but its tumor-promoting functions may prevail in later stages of cancer progression. In breast cancers (BrCa), the true functional impact of modulated NOTCH signaling remains particularly controversial and complex. It now appears that most NOTCH mutations found in BrCa represent GOF mutations, often linked to a simultaneous loss of hormone receptor functions [39]. This is specifically the case in the aggressive and often treatment-resistant triple-negative (TNBC) and basal-like carcinomas of the breast [40]. The aggressive behavior of these cancers may be further promoted by concomitant BRCA1 or BRCA2 mutations, cooperating with Notch signaling towards cancer progression. In BrCa, GOF Notch mutations and activation of canonical Notch signaling appear generally linked to increased aggressiveness, promotion of cell motility [41], EMT [42,43], and radiation [44] or chemotherapy resistance [45]. Tumor suppressors frequently mutated and lost in carcinomas, like PTEN (Phosphatase and Tensin Homolog), are also often functionally linked to GOF Notch signaling activities, as described for gastric cancers [46]. Their loss may result in a net activation of Notch signaling. A growing body of evidence further hints to the key role of several microRNAs, such as miR-182, as key mediators between altered Notch functions and cancer progression [47]. Similarly, loss of miR-449a was found to promote colon carcinogenesis, again linked to NOTCH activity [48], and miR-195-5*p* regulates *NOTCH2* expression in colorectal cancer [49]. Blocking NOTCH signaling by a γ-secretase inhibitor (GSI) duly results in enhanced radiosensitivity in two BrCa cell lines (MCF7 and T47D) [50]. Activating Notch mutations are also prominent in inflammatory BrCa, which show particularly poor patient outcome [51]. Generally, some of the most well characterized Notch-downstream genes may also exert the most prominent GOF effects, such as SOX9, HES1 [47], or HES5 [52].

### 3.2. Loss-of-Function NOTCH Mutations in Cancers and Its Consequences

There is mounting evidence supporting that loss-of-function (LOF) Notch mutations are strikingly predominant in epithelial cancer types or carcinomas, including squamous skin carcinoma and basal-cell carcinomas [53], pancreatic [54], liver (via *HES5*, [52]), and bladder cancers [26]. Functional inactivation or LOF of Notch-signaling can also reduce expression of critical Notch-regulated genes like FBXW7, which acts itself as a potent tumor suppressor [55]. The involvement of Notch mutations, and modulation of Notch signaling, appears particularly significant in squamous cell carcinomas, e.g., of the lung (LSCC), the head and neck (HNSCC, [56]), the oesophagus [57,58], and the skin (cutaneous squamous cell carcinoma) [59]. NOTCH1 (and probably NOTCH3 and NOTCH4) may be required for terminal or squamous differentiation of epithelial keratinocytes. Notch activity in keratinocytes may thus be one of the outstanding hallmarks of “squamousness”, the common features observed in these functionally similar epithelia, which are at least partly retained in the epithelial cancer types or carcinomas that are derived from these [60]. In this context, it is not surprising that squamous cell carcinomas from different tissues show striking histological similarities, indicating common differentiation patterns. This may be in line with the observation that inactivating mutations in NOTCH1, or loss of NOTCH1 due to deletions, occur early in the development of cutaneous squamous cell carcinomas [61]. Corresponding genetic events also predispose the oro-pharyngeal epithelium for tumorigenesis [62], and may therefore be outstanding early drivers of cancer initiation and progression. Specifically, NOTCH1 may play a dual role as either a tumor suppressor or (more rarely) as a protooncogene, specifically in squamous carcinomas. The true functional consequences for each Notch mutation found in a patients’ tumor cells remain poorly understood and likely depend not only on the oncogenic nature, e.g., of a certain NOTCH1 point mutation, but also on the mutational context of the tumor and its remaining lineage-specific, in this case epithelial, differentiation potential, while not even speaking of the interaction with the TME and the ECM.

Generally, there is also a strong link between altered Notch functions (typically, LOF) and the TME [63]. This is again a prominent hallmark in epithelial cancers, even more in squamous cell carcinomas. This characteristic feature likely indicates once again the strong functional link observed between Notch functions (specifically NOTCH1 and 3) and keratinocyte differentiation, a reminiscence of Notch signaling as a key pathway that promotes epithelial tissue morphogenesis, differentiation, and maturation. The loss of Notch’s differentiation-supporting functions has the potential to induce early stage skin carcinogenesis [53]. It also strongly impacts on the integrity of the stromal microenvironment of the resulting squamous carcinomas. Similar associations between Notch and alterations in the stromal TME are observed in adenocarcinomas, such as in colon cancer progression [64]. This almost ubiquitous strong correlation between Notch and the integrity of the stromal microenvironment may contribute to the frequently described induction of EMT in several cancer types, such as lung cancer [65] (see chapter 4).

### 3.3. Ambivalence of NOTCH Signaling in Cancer Initiation and Progression

The question whether NOTCH receptor mutations result in gain or loss of Notch functions is largely mediated by the activity of Notch downstream response genes, which is often poorly understood. We will specifically address the modifying role of Notch signaling on EMT via such downstream effectors in Section 4, which has also been reviewed elsewhere [66]. The complex interactions between Notch and up- or downstream regulatory mechanisms often make it difficult to understand the true functional impact of *NOTCH* mutations and altered Notch signaling in the context of cancer cells and tissues. For example, in glioma, NOTCH can act as both tumor suppressor and oncogene (reviewed in detail in [29]). Oncogenic, activating Notch mutations recently identified in glioma may specifically promote and maintain the stem-cell characteristics of glioma cells, the most aggressive type of brain tumors in humans. However, Notch-inactivating LOF mutations have also been found in glioma and other tumor types emerging from the forebrain region [67]. In this case, Notch exerts tumor suppressor activities, especially the NOTCH1 and NOTCH2 receptors, and functional Notch-mediators including RBPJ. To add to this controversy, genetic activation of the Notch pathway has been found to reduce glioma growth, and to increase patient survival, which may correlate to tumor subtypes and lower-grade tumors. This bi-directional role is also supported by analyses of the complex genetic and mutational landscape of gliomas [67] and their staggering clonal heterogeneity [68]. These features are general hallmarks of aggressive tumors, including advanced, metastasizing carcinomas. Again, it is mainly the context within the tumor tissue that matters with Notch functionality.

It appears that LOF of Notch signaling may specifically promote progression in some, predominantly epithelial cancers or carcinomas, possible also at early stages and even tumor initiation. This can be explained by the loss of regulatory control of Notch over epithelial pattern-formation processes, with a net growth advantage of cells suffering such mutations. Further tumor progression, which often includes acquisition of invasive/aggressive properties and EMT, may then be more frequently associated with Notch GOF mutations, similar to reports on stem cell maintenance in some cancer types like BrCa [69]. This GOF associated with tumor progression is supported by the characterization of oncogenic mutations in NOTCH receptors in cancers such as colorectal carcinomas (reviewed in [70]), breast cancers [71], and many cancer cell lines (Figure 1). These data indicate that differential activation or inactivation of NOTCH receptors, ligands, and Notch downstream genes may be involved at different stages or progression and in different microenvironments. Notch signaling may also change its mode of cooperation with other signaling pathways during progression. Distinct steps of progression, which can involve either activating or LOF mutations, may thus be highly characteristic and distinctive for various tumor entities and tumor subtypes, as well as for their specific stages of progression and acquiring an aggressive phenotype. It is now believed that modulation of Notch signaling contributes to the increasing heterogeneity observed in advanced and specifically metastatic cancers [63], itself a hallmark of cancer progression and poor patient outcome. This unique wiring of Notch signaling in a patients’ tumor may further promote the vast individual differences observed between cancer patients [72], which increases in advanced cancers. Thus, our understanding of Notch signaling and its impact may be critical for future personalized medicine [73]. We have also seen that increased, persistent Notch signaling is likely to be relevant for maintaining stem-cell characteristics of CSCs [74] and may thus contribute to tumor relapse after therapy, also known as “tumor dormancy”, or the persistence of occult micro-metastases, one of the main problems in current cancer therapy that is leading to poor patient outcome and early deaths. Better understanding of tumor relapse, therapy failure, acquired drug resistance, and subsequent metastasis are considered the holy grail of personalized medicine. In this context, there is also a massively increased interest in exploring the true role of Notch signaling for defining the “stem cell niche” in cancers [75].

### 3.4. Context Truly Matters with Notch: CROSSTALK with Other Signaling Pathways

**Figure 2:**
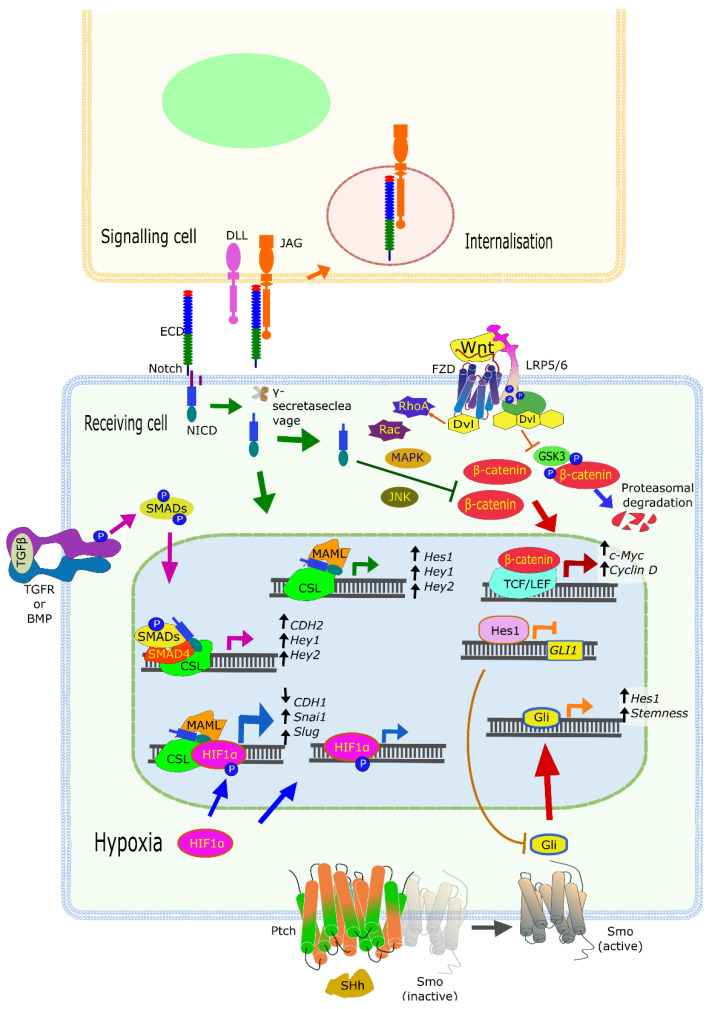
Notch crosstalk with other signaling pathways. Activation of Notch receptors by ligand-presenting cells results in the release of the Notch intracellular domain (NICD), forming a transcriptional activator complex with CBF1, Suppressor of Hairless, Lag-1 (CSL) and mastermind-like protein 1 (MAML). It also interacts with other signaling pathways, for example, by binding to β-catenin and inducing its degradation. The Wnt/β-catenin pathway is involved in cell cycle regulation. NICD also interacts with hypoxia-inducible factor 1-alpha (HIF1a) during hypoxia enhancing HIF1a transactivating activity. Likewise, SMAD transcription factors, which are activated downstream the TGFR and BMP pathways, also interact with NICD/CSL complex. Interactions with additional pathways include communication with Sonic Hedgehog (SHH), where binding to Patched (Ptch) results in the release of Smoothened (Smo) and activation of a cascade of signaling centered in Gli. Hes1, a downstream target of Notch, represses *GLI*, while Gli activation by Smo triggers downstream genes such as HES1 and other stemness genes. Gene symbols used according to Genome Reference Consortium Human Build 37 (GRCh37).

Increasing tumor heterogeneity, a hallmark of advanced, aggressive, and metastasizing cancers, relies on complex interactions of tumor cells with each other, but also with stromal, immune, and vascular cells. Most of the cell types that make up the TME also express various components of the Notch signaling pathway (including receptors and ligands). Maybe most importantly, they are simultaneously connected to multiple other signaling pathways altered in cancers. Here, we will describe some of the best understood, and most recently characterized Notch-related interactions between diverse cell types and signaling pathways in solid cancers, although this has been recently reviewed elsewhere [76]. Maybe the most important pathway known for crosstalk with Notch is the Wnt (Wingless) pathway (Figure 2).

WNT factors are a family of secreted proteins (at least 19 in humans) that act by binding and activating the Frizzled receptors (FZD). The 10 FZDs, all members of the superfamily of G protein-coupled receptor (GPCRs), are usually expressed in conjunction with membrane co-receptors, such as the Lipoprotein receptor-related protein (LRP)-5 or -6, or tyrosine kinase (TK) receptors. In short, the canonical WNT pathway involves the binding of WNT proteins to FZD and LRP5/6 factors, thus triggering phosphorylation of LRP, followed by recruitment of Dishevelled (DVL) proteins from the cytoplasm to the plasma membrane, eventually resulting in the inactivation of the β-catenin destruction complex. Activation of this central signaling pathway thus results in the stabilization and accumulation of β-catenin (CTNNB1), which translocates to the nucleus to interact with TCF and LEF family transcription factors (TCF7, LEF1, TCF7L1, and TCF7L2). The resulting transcription-activating complex switches multiple cellular processes on or off. Lack of β-catenin regulation leads to the transformation of specific subtypes of colorectal cancers, for example tumors carrying mutations in the adenomatous polyposis coli (APC), a member of the β-catenin destruction complex. This results in the accumulation of β-catenin and overexpression of downstream genes such as c-Myc and CyclinD1 [77,78,79]. These two factors are well-known drivers of cell cycle progression via the G1/S checkpoint, although β-catenin is also involved in other cell cycle events, i.e., by promoting centrosome cohesion [80].

In addition, WNT factors can also bind to FZD and ROR2 (Tyrosine-protein kinase transmembrane receptor 2). This activates the non-canonical β-catenin-independent WNT pathway, which also involves DVL proteins that activate G proteins of the Rho family (Rho and Rac, Figure 2) and Rho kinases (ROCK1 and 2). This, in turn, triggers intracellular Ca^2+^ fluxes and downstream Ca^2+^-dependent responses, e.g., reorganization of the actin cytoskeleton, and gene expression changes (reviewed in [81]). The promoting effect of Wnt/β-catenin signaling on cell cycle progression may be counteracted by Notch signaling, which activates a series of mechanisms that delay G1/S phase progression. This is achieved, for example, by direct binding of the NICD to active β-catenin, inducing the degradation of the latter [82]. Yet, none of this is straightforward, as a number of Notch-downstream genes play roles in promoting phenotypical changes that can result in tumor promotion. For example, HES1 actively represses the expression of p27 ^Kip1^, thus contributing to the proliferation of tumor progenitor cells [83]. These data suggest that Notch signaling can also function as a regulator of cell proliferation and division, alternatively stalling or promoting cell cycle progression, depending on other cell signaling pathways, the cell status, and the environment (especially within tissues).

Many of the findings outlined above raise the key question how Notch activity may sometimes act as a tumor enhancer and sometimes as a promoter? This can only be addressed in context, since Notch signaling relies on homotypic and/or heterotypic cell–cell contacts. Notch receptors and ligands may be expressed on the same cells, but also on different cells (cis- and trans-acting). With this strong emphasis on cell–cell communication, it is not surprising that Notch signaling plays a key role in the regulation and control of cell fate. Cell fate decisions are critical hallmark of differentiation or maturation of tissues, and their opposite: de-differentiation and acquiring stem-cell like properties. This is possibly most prominent in stem cell renewal, which helps cancer cells to maintain a stem-like phenotype (“stemness”) within a larger tissue-context. Stemness may promote long-term quiescence and survival of tumor cells in the corresponding stem cell niches, eventually resulting in tumor relapse long after (chemo-)therapies have ended. Quiescence and refraining from cell cycle progression are “traditional” hallmarks of stem cells, but this may be ambivalent in CSCs. Increased stem-cell characteristics may also promote a highly variable, but consistent proliferation potential to tumor cells, a less clearly understood and probably highly variable characteristic of CSCs that differs from non-transformed stem cells. Notch signaling is often activated in cancer cells that show enhanced stem cell maintenance. Notch-positive CSCs have recently been shown to be preferentially associated with endothelial cells within the tumor tissue, which is likely to act as a perivascular stem cell niche in breast cancer [84,85]. It was shown that tight association of quiescent cancer cells with an effective stem cell niche can significantly contribute to tumor dormancy and protect CSCs from therapeutic interventions such as chemo- and radiotherapy [84]. A similar perivascular stem cell niche for the persistence of CSC has recently been characterized in detail, and found to be promoted by active Jagged-1-Notch-1 signaling [84,85]. The Notch-dependent response in these CSCs involves the upregulation of ZEB1 in a positive feedback loop and stimulates endothelial cells by paracrine production of VEGFA. Disrupting the cycle, e.g., by ZEB1 deletion, decreases tumorigenesis and progression in vivo [85]. The mechanisms by which Notch signaling may promote stemness are certainly complex and may also involve additional cell types other than endothelial cells. For example, there may be functional involvement of stromal cell types (like fibroblasts and cancer-associated fibroblasts, or CAFs) with the potential to enhance stemness-supporting signaling loops. Mammary gland stem cells express DLL1 to activate NOTCH on stromal macrophages in vivo. In return, tumor-infiltrating macrophages respond to Notch-signaling by increasing the expression of WNT ligands (Wnt3, Wnt10A, and Wnt16 in particular) that feedback on CSC functions [86]. CAFs also promote stemness via secretion of IL-6, which activates the STAT3 signaling and the expression of Notch signaling molecules in hepatocellular carcinoma cells [87].

Another important signaling cascade linked to Notch is Hedgehog (Hh) signaling, a pathway (like Notch itself) that is active mostly during tissue and organ development. In the postnatal period, Hh signaling is restricted almost entirely to stem cells but can be re-activated in tissue repair and wound healing or inflammation. There are three Hedgehog glycoproteins (Sonic (SHH), Indian (IHH), and Desert (DHH) Hedgehog), of which SHH has been most frequently associated with cancer progression in several tumor types. SHH acts upon the 12-transmembrane proteins Patched (PTCH1 or 2, Figure 2), inhibitors of a constitutively active GPCR named Smoothened (SMO). This interaction results in the release of SMO from PTCH1 and triggers an intracellular cascade of events that eventually lead to the activation and nuclear translocation of GLI transcription factors [88]. As expected for anything related to Notch, the functional Notch–Hh interactions are complex and occur at different levels. HES1, a downstream target of Notch, binds and represses *GLI1* (glioma-associated oncogene 1) and regulates Hh signaling in melanoma cell lines and primary glioblastoma cultures [89]. It appears that Notch and Hh signaling regulate and sometimes compensate for each other, as *HES1* is also a target of SHH in stem-like cells [90]. The crosstalk between Notch and Hh, as well as Wnt, results in compensation when one of these pathways is suppressed. For example, Notch inhibition (using GSI) results in upregulation of Hh and Wnt signaling activity in glioblastoma cells [89]. Targeting both Notch (with GSI) and Hh (by cyclopamine) induced cell death by apoptosis and blocked colony formation in comparison to either inhibitor alone, thus confirming the nature of compensatory signaling cascades. Tumors in advanced cancer patients with a hyperactive Notch–Hh signature also show increased stemness properties. Such cells were again found in niches with generally low, or hypoxic, oxygen content. These cells are also immune-privileged by attracting immunosuppressive T-cells, myeloid-derived suppressor cells, and tumor-associated macrophages [91]. Altogether, the balance and synergies between these signaling pathways, and many others such as EGFR and BMP, determine the outcome of cell fate instructions by Notch signaling. They may also be responsible for the ambivalent nature of Notch pathway activation versus inactivation in many cancer-relevant processes.

### 3.5. A “Holistic” Approach: NOTCH-Related Signatures, Gene Sets, and Pathways

Since 2004, our understanding of Notch deletions and amplifications, point mutations causing either gain or loss of functions, but also differential expression and activity of Notch ligands and downstream executors has yielded a large body of evidence and over 7500 publications covering almost any cancer type. However, it has also become increasingly evident that genetic alterations in the four human NOTCH receptors and five ligands may only represent the tip of the iceberg. Many additional genes are linked to Notch signaling, either as upstream regulatory factors, or down-stream effectors and executors of Notch functions. Many of these genes are themselves mutated at low or very low frequencies in cancers. Such rarely mutated genes have been termed “long tail” cancer genes: They represent a long string of many genes (the “tail”) that are significantly affected as a whole, but only emerge more prominently when large numbers of cancers are analyzed. Such “long tail” genes may be mutated with low penetrance as the result of slow, stochastic processes in which each candidate provides only a small growth advantage for cancer initiation and progression. Each mutation by itself may not have the potency to significantly shift the balance towards tumor progression, but many such genes combined do. Which of the genes are affected in a patients’ tumor depends on random genetic events, followed by slow Darwinian selection in the emerging pre-malignant, later malignant cancer tissues. For example, such small and incremental advantages may only result in tiny increases in cell proliferation and protection from apoptosis, slightly more successful evasion of immune cells, and later also to moderately increased resistance to anti-cancer drugs. Such genes may provide tiny benefits for cancer stem cell survival, homing at the stem cell niches, and tumor dormancy. Together, however, they may be critical for overt tumor progression and relapse at later stages. For example, a large number (>400) of such “long tail” cancer genes with relevance for Notch pathway modulation have been identified in HNSCC [92]. Our own analyses in databases confirm the existence of such “Notch fingerprint” genes in HNSCC and other tumor entities. The functional relevance of some candidate genes, such as AJUBA, for in vivo cancer initiation and progression has subsequently been validated by functional CRISPR-Cas9 screens [93]. Research on such prognostic gene signatures, related to Notch (and EMT) in cancers, is only beginning. In total, these new “systemic” data indicate that even rare mutations, in combination with the more frequent mutations observed for the four NOTCH receptors themselves, may contribute to progression and carcinogenesis of two thirds (67%) of all HNSCC cases [92].

These and other Notch-related genes and gene sets can be validated by systematically exploring mutation frequency and/or over-expression in human genomic databases, such as the TCGA (The Cancer Genome Atlas), e.g., using the ICGC database tools for mining [94]. These data bases contain both genomic data (mutations, deletions, amplifications) and mRNA expression data (RNAseq, or older microarray data). It is likely that such “long tail” Notch-related gene signatures are strikingly different between cancer entities and subtypes, and even between patients with otherwise patho-morphologically similar tumors. It is also expected that there is significant overlap of “long tail” genes and their net activating effects with other cancer-relevant pathways [95], in particular the previously mentioned canonical and non-canonical WNT pathway [96,97], the HIPPO pathway [98], but also the AKT and PI3Kinase pathways [99] or K-Ras signaling [100]. Finally, there is also indication that distinct NOTCH receptors, such as NOTCH2, play distinct and specific roles in this overlap, e.g., with WNT signaling [101]. Only very recently, comparable distinct roles for NOTCH ligands in various cancer types and in connection with certain signaling pathways have been identified, such as for JAG1 [102], DLL1, and DLL4 [103]. We will see that there is a particularly high degree of interaction of NOTCH signaling (and many of the rare genes modulating Notch signaling) with processes that drive EMT, which can only sometimes be traced back to certain NOTCH receptors like NOTCH2 [102]. This high connectivity of NOTCH signaling as a whole is particularly interesting and important for future drug discovery and target validation approaches, and functional NOTCH modulator screens [104].

**Figure 3:**
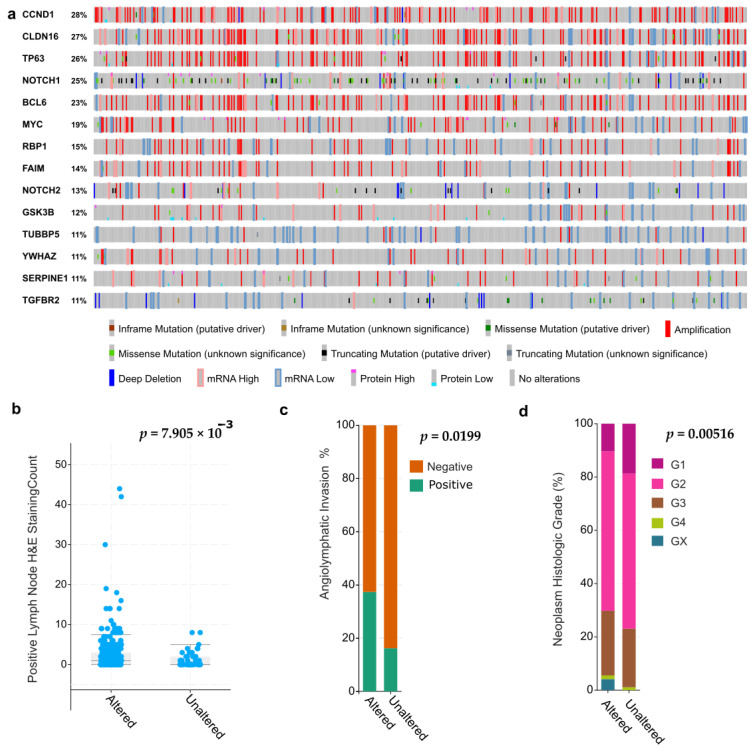
Analysis of most frequently altered NOTCH and EMT-related genes in head and neck squamous cell carcinomas (HNSCCC). (**a**) Mutation and gene expression profiles for 12 target genes associated with both Notch signaling and EMT in the cBioPortal genomic database (sorted by patient number, not mutations). Indicated are mainly genomic amplifications, deletions, and point mutations, followed by mRNA and protein overexpression across 530 HNSCC tumor samples from the “Firehose legacy” sequencing project. (**b**) Number of patients with positive lymph nodes (left) is increased for tumors that harbor genetic alterations in the 12 Notch/EMT genes show above. (**c**) Patients with a high degree of genetic changes in Notch/EMT signature genes also show a larger proportion of tumors with angiolymphatic invasion, or penetration of tumor cells into lymph vessels in the tumor periphery. (**d**) Patients with large numbers of Notch/EMT genes mutated further show a larger likelihood to develop high grade tumors (G3 and G4), with enhanced invasive properties.

**Figure 4:**
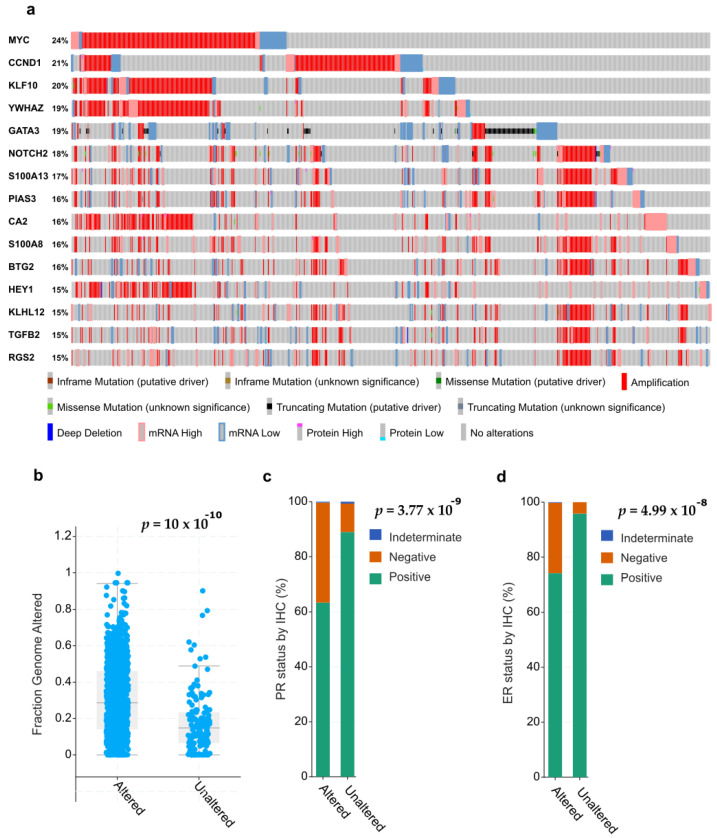
Analysis of the 12 most frequently mutated genes associated simultaneously with Notch signaling and EMT, in 1039 breast cancers (TCGA sequencing project). In contrast to Figure 3, tumors have been sorted according to most frequent mutations (CCND1, MYC) to illustrate the high frequency of amplifications. (**a**) Sorted incidence of mutations, including amplifications, deletions, point mutations, and RNA overexpression, as generated by the cBioPortal browser. Data have been sorted according to type of mutation/genetic alteration. (**b**) Tumors with high frequency of genetic alterations in Notch/EMT target or signature genes also show a higher degree of genetic instability. (**c**) Tumors with high levels of Notch/EMT-related genetic alterations more frequently belong to the triple-negative and basal-like breast cancers that lack expression of progesterone receptor (PR). (**d**) Corresponding finding for expression of estrogen receptor (ER) in the same tumors, as analyzed by immunohistochemistry (IHC).

We have identified mRNA gene expression patterns from very different cancer types and localizations that show striking similarities and overlaps, including common genetic events such as amplifications, leading to overexpression of the target genes. We show such representative patterns for HNSCC and BrCa in Figure 3 and Figure 4.

Other tumor entities can show different signatures, only partly overlapping to those shown in Figure 3 and Figure 4, although often also very prominent and with high frequency of mutations (typically, >20% of tumors). A large number of genes associated with Notch signaling have been extracted from comprehensive gene expression databases, such as the MSigDB database at BROAD Institute/MIT [105]. These genes (over 1000) were explored for genetic events (mutations, amplifications, homozygous deletions) and differential mRNA expression in large numbers of human tumors, using the cBioPortal browser [106] that provides open access to the TCGA collections of genetic analyses of primary cancers; Figure 3 and Figure 4).

The results from some of our bioinformatics analyses point to remarkable similarities in the mRNA expression patterns and frequency of genetic events affecting similar panels of Notch-related genes, including the 4 NOTCH receptors themselves, between different tumor types. This is particularly striking between squamous cell carcinomas of the head and neck (HNSCC) and the lung (LSCC, not shown). These strong similarities indicate a superior impact of tissue origination, morphogenesis, and intrinsic differentiation patterns for gene expression patterns observed in certain cancer subtypes (in this case, the squamous cell carcinomas of different localizations), and Notch-related genes. This may reflect functional similarities (such as the previously mentioned lineage-specific developmental programs) in the corresponding cells of origin, as a hallmark of the common “squamousness” of these normal tissues. This may result in similar oncogenic processes, and similar panels of genes affected by genetic alterations, that lead to tumor initiation and progression in these cancer entities (Figure 3). The origin of squamous cell carcinomas (and thus “squamousness”) are the keratinocytes, epithelial cells with strong mucosal differentiation potential found in the skin, the cervix uteri, the lungs, upper aerodigestive tract, and the oral mucosa. It is thus not surprising that skin, cervical, oral, and lung squamous cell carcinomas share several Notch-regulated or Notch-regulatory genes, which are frequently amplified and overexpressed, deleted, or show recurrent point mutations. We have further observed that these remarkably similar gene signatures may be further linked to both Notch signaling and EMT across the squamous carcinomas: over 180 genes have been identified in the MSigDB data base that are associated with both EMT and Notch signaling, many of which show a high percentage of genetic alterations (>10%) in HNSCC (Figure 3a). Such genetically conserved gene sets correlate with measurable phenotypic features such as lymph vessel invasion (angiolymphatic invasion), as shown for HNSCC (Figure 3b–d). Expression patterns of Notch and/or EMT-related genes are also detectable in other, non-squamous tumor types, such as lung adenocarcinomas (tumors with glandular differentiation patterns), but they are strikingly different (Figure 4a). For each tumor type or subtype, characteristic sets of Notch/EMT-associated genes or “gene signatures” can be identified, which often correlate with advanced histopathological features and more aggressive tumor subtypes. This is particularly prominent in TNBC and basal-like BrCa, which simultaneously lack expression of oestrogen receptor (ER), progesterone receptor (PR), and HER2 (Figure 4c,d). Altered Notch/EMT genes were also associated with increased genomic instability and mutation load (Figure 4b). The association of Notch mutations with TNBC is not novel, and has been independently observed and confirmed in several studies [40,107]. In this fashion, cell- and tissue-specific epithelial differentiation and hormone-regulated maturation, e.g., in BrCa, are opposed to de-differentiation, concomitant with increased tumor cell aggressiveness or invasion, EMT, and (possibly) metastasis. These phenotypes represent the extreme ends of a spectrum of processes linked to Notch functionality. It remains, however, unclear which genetic alterations in Notch signaling occur first and what are their functional consequences for cancer initiation and progression. To tackle the complexity and chronology of events, it may be highly beneficial not to work only with single genes (like NOTCH receptors 1–4) or investigate functional consequences of the genetic changes that are affecting them. Instead, it may be worthwhile to use a broader, “holistic” approach and explore the activity of Notch together with the functionally linked pathways, gene sets, or gene signatures. This can be done across many individual patients’ tumors, hopefully revealing critical insights into (expanded) Notch functions for clinical decision-making and personalized medicine.

### 3.6. Functional Validation of NOTCH Signaling and Personalized Cancer Medicine

The direct consequences of Notch receptor mutations, but also of many rare mutations in Notch-associated genes and pathways, have not been systematically validated. We still do not know the true function of many or even most of these genes, and the due consequences of genetic mutations in the context of living cancer tissues. To a large degree, this even applies for the functions of the NOTCH1-4 receptors and ligands themselves. It is therefore not surprising that no Notch-targeted drugs have been approved yet for any anti-cancer therapies (although drug discovery efforts continue). Over the past 10 years, different classes of drugs therapeutically targeting Notch including receptor/ligand antibodies have been clinically tested, most of them gamma (γ)-secretase inhibitors (GSI). Only more recently, a new class of Notch transcription complex inhibitors has been developed, including SAHM1, a circular peptide that targets the protein–protein interface and prevents Notch complex assembly [108]. Another novel type inhibitor is FLI-06 [109] which disrupts Notch trafficking and processing. A particularly promising novel type Notch inhibitor is CB-103, which directly targets the NOTCH transcriptional activation complex [110]. New pharmacological developments concerning the specific inhibition of Notch are summarized in [111]. In the year 2020, over 70 cancer clinical trials have been registered, typically early phase I and II trials. Nevertheless, at least one clinical study phase III currently recruits patients for a trial on the drug *Nirogacestat,* another GSI. Time will show if any of these trials are more successful than those of the past, and one can hope that better functional insights may promote the success of early-stage drug discovery, and clinical evaluation.

Our lack of functional understanding is also due to our consistent lack of model systems to explore Notch functions in cancers. Much of Notch-related research has been performed in animal models that are of limited value for cancer research, as mainly human cells (cancer cell lines) are utilized, e.g., for xenografts, which rarely metastasize, e.g., in mice. There is also a severe lack of useful in vitro model systems, which would be complex enough (and yet standardized) to allow investigating the impact of NOTCH-mutations observed in solid cancer tissues. Current models make it very difficult to quantitatively measure their potential impact on progression and invasion. However, it should be possible to explore the interactions, e.g., between cancer and stromal cells (such as cancer-associated), provided they recapitulate the connectivity of NOTCH receptors and ligands. However, few in vitro models have been explored for this research purpose. As a consequence, there is relatively little research related to the true impact of Notch receptors, ligands, and pathway(s), especially in the clinically most relevant processes such as tumor cell invasion, lymph-angiogenesis, lymph node, or distant metastasis. Similarly, we lack suitable, standardized cell culture and/or tissue models to explore the role of Notch signaling in formulating the tumor stem cell niche and tumor dormancy, and how this may affect the emergence of drug resistance and relapse. Considering the ambivalence and partial redundancy of Notch, it is questionable if genetic analyses of genes like the NOTCH receptors 1–4 by themselves will contribute significantly to predicting patient outcome, response, or resistance to therapy. Without models systems with matching complexity, it will also remain difficult to experimentally address altered Notch functions in the context of the TME, and its interactions with e.g., CAFs, endothelial cells, or invading immune cell populations. Similarly, Notch signaling is likely to be affected by changes in the nature and density of the ECM, which is critical for mechano-sensing within tissues. The nature of the ECM in cancers is surprisingly difficult to mimic in vitro, and most model systems use artificial, poorly characterized and non-human ECM extracts such as mouse Matrigel, or pure collagen; none of these are close proxies for the ECM in living tumor tissues. In addition, it is difficult to achieve not only the complexity, but also the high density of the ECM observed for tissues in model systems.

Apart from the NOTCH receptors and ligands themselves, and the pathways they engage in serious crosstalk with, a growing number of additional, somatic drivers of cancer progression were identified that may regulate, or are regulated by, altered NOTCH activity [112]. Such studies are rare, but will be necessary to assign reliable proto-oncogene or tumor suppressor gene-functions to such rare targets. It is promising that some of the more prominent and widely researched Notch target genes such as HEY1 have been identified as prognostic and diagnostic biomarkers. Elevated expression of HEY1 indeed correlates with poor patient outcome [113] in HNSCC. Differential activity of the Notch pathway and downstream signaling may also be functionally linked to the differences observed between Human Papillomavirus (HPV) positive and negative HNSCC [114], a key question for treatment of patients. It is possible that NOTCH1 mutational status promotes the development of (HPV)-associated oral or cervical cancers. The additional HPV oncogenes like E6 and E7, with their potent modification of the retinoblastoma (Rb) and p53 (TP53) regulation of the cell cycle progression, may disrupt or modulate the tumor-suppressive activities of Notch signaling, and vice versa; links between Notch and target genes like *Cyclin D1* and *MDM2* may also contribute to these net effects, as has been outlined above. Notch and cell cycle progression pathways may exert a mutual influence on each other. The net effect may then have consequences for tumor progression (HPV^+^ tumors rarely metastasize) and the response to therapy (HPV^+^ tumors respond better to therapy, rarely metastasize, and show better outcome) [38]. NOTCH mutations and NOTCH-related pathway activities, or gene signatures, also have an impact on many other epithelial cancer types, including colon- or colorectal cancer (CRC) [115] with a specific impact on the stemness and mesenchymal properties of these cancers [116], possibly due to the strong functional links to Wnt/β-catenin signaling outlined above. In CRC, Notch-mutations and pathway activities may also be specifically linked with recurrent KRAS mutations [117]. This can be blocked by recombinant antibodies targeting the DLL4 protein at the cell surface of cancer and/or stromal cells within these tumors [117], or possibly the endothelial cells in these tumors that also express DLL4.

## 4. NOTCH and EMT Signatures

The basic ability of epithelial cells to trans-differentiate into a more mesenchymal-like phenotype can also be acquired (or hijacked) by tumor cells. One of the most distinctive features of the EMT is the loss of E-cadherin (*CDH1*) expression, which leads to the loss of tight cellular junctions and epithelial polarization and enables increased motility. With the epithelial integrity lost, it becomes easier for tumor cells to engage in cell motility and migration or invasion. The regulation of EMT is still a matter of very active research, which continues to deliver surprises. EMT profiles can be highly variable, dynamic, and transitional, even within the same tumor, due to heterogeneity and high levels of genomic instability [118]. Nevertheless, it has been clearly demonstrated that EMT promotes processes like cancer invasion and fibrosis [119], which are intricately associated with cancer progression. It remains unclear, however, if EMT also promotes metastasis, in particular distant metastasis. EMT may indeed contribute to local invasive procedures, which are at the same time highly relevant for clinicians (e.g., the definition of positive tumor margins, and the “budding” of tumor cells into the vasculature). EMT may thus also promote or favor the penetration of lymph and blood vessels and the formation of lymph node metastases. However, successful metastasis of “seeds” (=tumor cells) to a fertile “soil” (=at a distant metastatic site) may require more than just an active cytoskeleton and invasive properties. We will see in the following paragraphs that EMT itself is delicately balanced, and it may in fact be part of this tight balance, which also drives successful dissemination of tumor cells to distant sites.

NOTCH1 activation is further known to repress *CDH1* expression by complexing with CSL (now RBPJ) and the *CDH1* promoter [120]. The same molecular machinery is used to activate expression of the Snail1 (*SNAI1*) and *Slug* (*SNAI2*) transcription factors, well-known and central EMT markers and regulators [121,122,123] of outstanding relevance. Both transcription actuators have been reported to further repress *CDH1* and induce cell invasion [43]. In animal models of ectopic and orthotropic colorectal cancer, infiltrating Jag2-expressing bone marrow cells were found to induce EMT in cancer cells, by a Notch-depended decrease of E-cadherin and concomitant increase of vimentin expression [124]. Moreover, mesenchymal factors seem to be able to fuel Notch signaling. For example, the EMT-inducer Twist1 (TWIST1) may enhance Notch activity by increasing the expression of “classic” Notch-downstream genes (*HEY1*, *HEY2*, and *HES1*), but not the Notch ligands or receptors [57]. TWIST seems to also play a pivotal role in tumor progression [125]. A number of studies show that TWIST overexpression is strongly associated with cancer invasiveness and metastasis in BrCa in both animal models [126,127] and in patients [128]. Similarly, a functional knock-out of *TWIST1* inhibits cell plasticity and metastasis [126]. For example, in glioblastoma (and possibly other tumor types), the glycoprotein Epsin3 (EPN3) may be related to Notch and WNT/β-catenin signaling pathways, and helps to induce EMT in glioblastoma cells after activating SLUG, TWIST, and ZEB1, but not Snail1 or ZEB2. Experimental data further indicate that EPN3 may promote the migration and invasion of glioblastoma cells [129]. We cannot refer here to a plethora of newer reports related to EPN3/Epsin functions, e.g., in podocyte formation, invasiveness, and tumor cell plasticity. In contrast, recent studies have shown that miR-139-5*p* has been defined as a tumor suppressor that inhibits *NOTCH1* and restrains metastasis and EMT of glioblastoma [130].

Notch activation of EMT is further strongly enhanced under hypoxia, where hypoxia-inducible factor 1α (HIF-1α) complexes with CSL, NICD, and MAML, resulting in higher *SNAI1* expression [120]. This relationship has been confirmed in many cancer entities, including pancreatic cancer, bladder cancer, as well as oral squamous cell carcinoma [131,132,133]. HIF-1α also induces EMT by overexpression of lysyl oxidase (LOX), which in turn stabilizes Snail1. Yet, NOTCH1 can substitute a blockade on hypoxia and trigger EMT in squamous cell carcinomas (SCC). Additional data show that Notch signaling is essential to link the hypoxic stimulus with induction of EMT, thereby inducing progressive motility and invasiveness [134]. Notch-ligand activation by hypoxia was found to stimulate Jagged 2 in BrCa cells, trigger EMT, and enhance cell survival in vitro [135]. Moreover, studies demonstrated that the Notch ligands Jagged1, DLL1, DLL3, and DLL4, except Jagged2, were all expressed in mesenchymal stem cells (MSC), and strongly increased by HIF [136]. The HIFs may thus also control molecular signaling between BrCa cells and MSCs, as a means to stimulate invasiveness and possibly metastasis [137]. High expression of NOTCH1 was also observed in organoids from SCC, which exhibit EMT features, suggesting the association of this pathway with mesenchymal properties [138]. Indeed, blocking of Notch signaling decreases the expression of EMT markers, and inhibits cell migration in many different types of cancer, such as oral squamous cell carcinoma, CRC, and BrCa [122,134,139]. The inhibition of Notch signaling in liver cancer resulted in differentiation of liver CSCs into mature hepatocytes via the opposing process to EMT, termed mesenchymal–epithelial transition (MET). This very same process also resulted in reduced malignancy [140]. Moreover, simultaneous inhibition of SMAD4 and NOTCH1 led to reduction of EMT activities in cancer cells [118]. In vivo mouse models have further shown that deletion of *NOTCH1* forcefully decreased mouse xenograft tumor formation [138].

Like the NOTCH receptors, Notch ligands are also related to the induction of EMT in cancer. Elevated expression of JAG1 correlates with poor prognosis in many cancer type [85,141,142]. Notch activation via both DLL and JAG ligands leads to induction of EMT, and interaction with Jagged increased the formation of cell clusters, a specific phenotype that could be stabilized by Numb or Numbl proteins [139,143]. It was further shown that JAG1 is a substrate of K-ras signaling in CRC [141]. While Notch ligand–receptor interactions are rarely investigated in experimental studies, it has been confirmed that JAG1 may be critical for Notch/KRAS signaling and required to promote tumor aggressiveness [141]. The conditions in the TME, such as hypoxia, and the presence of transforming growth factors or cytokines may further affect the fate of cells. Under these conditions, NOTCH1 signaling may be modulated by physical interactions with different transcription factors, which can lead to activation of non-canonical signaling pathways. The overexpression of ERα and resulting estrogen effects may also activate Notch signaling, e.g., via binding to the *NOTCH1* promoter in prostate cancer cells. As a result, the increased of NOTCH1 expression enhances EMT [142]. It is also known that cross-talk between Notch signaling and Transforming Growth Factor beta (TGFβ) is critical for induction and maintenance of EMT [118]. TGFβ signaling by itself is known as one of the most potent regulators of epithelial differentiation and EMT and (like Notch) is also a key driver of developmental processes. The presence of TGFβ in the microenvironment strongly induces normal squamous cell differentiation. In tumors, however, TGFβ may induce a shift in the spectrum of NOTCH1 target genes, promoting EMT. In addition, NOTCH1 and ZEB1 may cooperate to promote EMT in the presence of TGFβ [138] (Figure 2). The analysis of the *TGFβ* promoter shows a number of canonical CSL (RBPJ)-DNA binding motifs, which play a key role in Notch signaling [64]. There is also known crosstalk between bone morphogenic proteins (BMPs) and Notch to promote EMT, in which BMPs activate Notch via SMAD proteins, in a γ-secretase independent manner (non-canonical Notch signaling) [144]. BMPs belong to the TGFβ superfamily, with overlapping downstream signaling; therefore, this connection may not be surprising. The BMP/Notch link appears specifically relevant for colorectal and other cancers with glandular differentiation of the adenocarcinoma type [144], in which BMPs (via EMT) may specifically promote invasive properties. Malfunctions and “driver” mutations in NOTCH1 can also exhibit oncogenic properties, for example through activation of the epidermal growth factor receptor (EGFR)-phosphoinositide 3-kinase (PI3K)-protein kinase B (AKT) signaling pathway [99,145], usually described in short as the PI3K/AKT/mTOR pathway.

The interaction between signaling from different NOTCH receptors in subtypes of cancer differs significantly. In contrast to NOTCH1, NOTCH3 limits EMT in squamous cell carcinoma [138]. In BrCa lines (MCF7 and SUM149), NOTCH3 expression is activated by hypoxia via HIF-1α. NOTCH3 in these BrCa lines downregulates expression and secretion of the pro-inflammatory cytokine IL-6 via HEY2 expression. When *NOTCH3* was knocked out, these lines grew more steadily under constant stimulation by IL6 and downstream STAT3 activation. Blocking IL-6 has the opposite effects, and even potentiates the anti-tumor effects of GSI in NOTCH3-expressing BrCa cells. Expression of IL-6 also affects the proliferation of the mesenchymal-stem cell population (MSCs, described as CD24^-^/CD44^+^ phenotype) [146]. These findings suggest that NOTCH3 can play, at least partially, opposing roles relative to NOTCH1, especially in regulating EMT and stemness, and under these specific cell culture conditions and cell lines. Like every good actor, NOTCH receptors therefore show an outstanding degree of flexibility and readiness for complex interactions with other actors. In a liver cancer model, the knockdown of NOTCH1, NOTCH3, and NOTCH4 led to an increase of *CDH1* expression, with due consequences for EMT, while NOTCH2 had the opposite effect [118]. Additionally, posttranscriptional regulation of NOTCH2 through miR-195-5*p* in colorectal cancer leads to a significant reduction of NOTCH2 protein, and subsequently to a lowered EMT profile [49]. Blocking of ligand activation of Notch (all receptors) by using a soluble Notch4 exodomain (XNotch4) in BrCa xenografts promoted apoptosis and reduced tumor size and metastasis concomitantly to *SNAI2* expression reduction, *CDH1* upregulation, and active β-catenin suppression [147]. The non-canonical Notch activity, which does not require γ-secretase cleavage, results in a variety of not yet well-characterized interactions such as inhibition of the potent tumor suppressor PTEN, which leads to the activation of PI3K-AKT-mTOR survival axis [13]. AKT also play roles in cytoskeletal changes that result in changes in cell migration [148].

## 5. Notch in Cell Migration/Invasion

The involvement of Notch signaling has been reported already at relatively early stages of cancer spread, namely invasion and migration. To invade, epithelial cancer cells have to penetrate the ECM, or more specifically, the basement membrane/basal lamina, which delimits epithelial tissues. Some studies show that Notch, whether directly or indirectly, interacts with components of ECM (summarized in a recent review [149]). For example, Notch receptors can directly bind to ECM glycoproteins like CCN3 (Cellular Communication Network Factor 3) and TSP-2 (Thrombospondin 2), which may facilitate or block the interactions with Jagged ligands and influence the activity of Notch signaling pathway. A potential functional connection between NOTCH1 and Thrombospondin has also been described for hepatocellular carcinoma [150]. It was observed that Notch1 may specifically bind to soluble E-cadherin (sE-Cad) and Trombospondin-1 (Thbs) and that these proteins are released from tumor cells when Notch1 is targeted. Additionally, ECM components like the laminins, key components of the basement membrane, were shown to modulate the transcription of Notch receptors and ligands. Although brain tumors like glioblastomas and neural-type cancers like neuroblastomas rarely metastasize, they are among the most aggressive tumors. In these locally invasive cancers, enhanced Notch signaling might fuel their aggressive potential, as suggested by recent studies on mice. Blocking laminin expression in brain tumors leads to a decrease in expression of Notch ligand DLL4 at the protein level, decreasing the tumor volume [151], although this may primarily relate to the critical functions of DLL4 in the tumor vasculature in general, and endothelial cells in particular. Glioblastoma stem cells with downregulated NOTCH1 not only had a reduced self-renewal potential but were invasion-deficient in both the cell lines and mouse xenografts. Further detailed experiments revealed that low Notch signaling is unable to stimulate the expression chemokine receptor CXCR4, which in turn cannot trigger the AKT/mTOR pathway essential for cell invasion [152].

The mechano-sensing properties of Notch are regulated in a variety of ways. The first level of regulation occurs by the type of ligand that binds. This process may differentiate between the frequently observed shifts between pulsating versus constant Notch activation by different ligands [153]. This may relate to the ON/OFF switching of Notch signaling that requires the interaction of two ligands, the second ligand for permanent activation. This phenomenon may also define the binding strength of ligands to the Notch extracellular domain [154]. In addition to the ligand type, the characteristics of the “sending cell” matter. It is relevant whether such cells have more mesenchymal properties (indicated by co-expressing vimentin, VIM, and Jagged), which increases signaling strength. In contrast, VIM and DLL ligands seem not to cooperate and show no additive effects [155]. The successful invasion of adjacent tissues by tumor cells often involves the secretion of matrix-degrading proteases, in particular matrix metalloproteases (MMPs). High level expression of MMPs can also be a typical feature of EMT, and therefore, a link to Notch signaling would be interesting. Indeed, NOTCH1 signaling can augment the transcription of MMPs, in particular MMP2 and MMP9. This occurs likely indirectly via NF-kB activation [156]. Knock-down of NOTCH1 results in a concomitant decrease of MMP9 expression in prostate cancer cells [157], and of MMP2 and MMP9 in BrCa cells [158].

**Figure 5:**
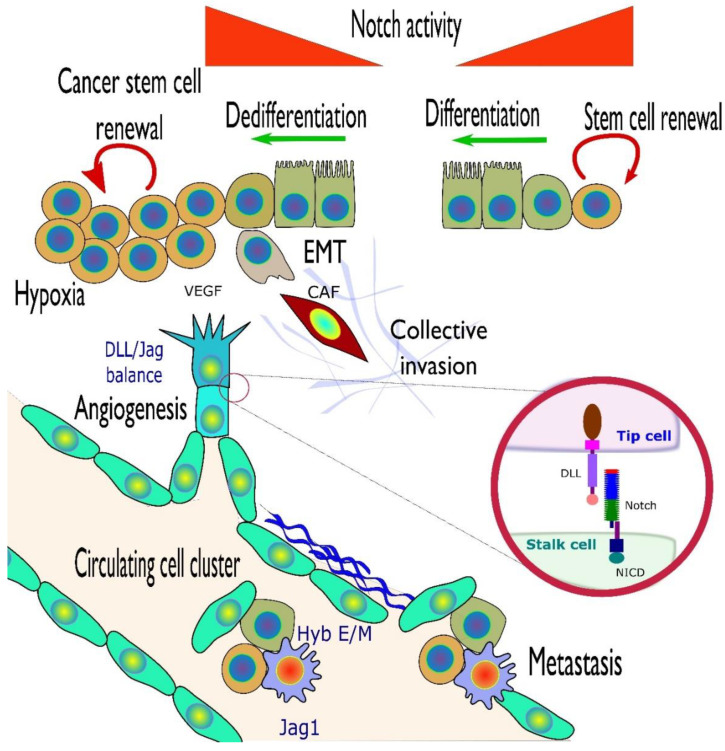
Notch activity in different contexts, exemplified here within the context of epithelial tissues and cells (olive-green cells, partly with brush border). Notch activity is important in stem cell renewal and differentiation (top-right). This is hijacked in some cancer cell types to maintain stem-like features such as unlimited proliferation and acquiring resistance to radio/chemotherapy (cells orange-brown color), in others to gain a mesenchymal phenotype (gray cells) that can resemble the phenotype of cancer-associated fibroblasts (CAFs, dark brown) and may result in collective invasion, characteristic for epithelial cells. As tumors grow, they may become increasingly hypoxic, which triggers neo-angiogenesis. The vasculature (endothelial cells, turquoise) is positive for Notch receptors and ligands, thus the interaction of the vasculature with tumor cells is likely a factor in invasiveness. One of the most striking features of Notch signaling, via Jag1 and hyb-E/M (hybrid epithelial-to-mesenchymal transition), is its supportive role in circulating clusters of tumor cells that have particularly high metastatic potential (olive colored, rounded cells), partnering also with immune cells (stellate, blue cells with red nuclei). The inset shows the ligand–receptor activation between 2 cells. Gene symbols explained in the text.

After breaking through the laminin-reinforced basement membrane (BM) and the ECM, cancer cells can invade blood and lymph vessels (illustrated in Figure 5). This process is termed intravasation and relies on the direct interaction of cancer cells with macrophages, podocytes, and endothelial cells. Upon this interaction, cancer cells form actin-based structures called invadopodia, which aid the local migration and invasion processes. These invadopodia or podosomes (now increasingly summed up under the term “invadosomes”) are mostly observed in cell culture but are thought to correlate to similar invasive structures observed when tumor cells locally degrade the ECM. Díaz et al. report that Notch signaling mediates activated invadopodia formation upon hypoxia. This occurred by ADAM12-dependent release of Heparin-binding EGF-like growth factor (HB-EGF) and subsequent activation of epithelial growth factor receptor (EGFR) in various types of cancer, including head and neck, lung, and pancreatic tumors [159]. The assembly of invadopodia was found to be coordinated by the Mena^INV^ protein, which is activated upon macrophage-NOTCH1 contact. This was shown in BrCa cells and subsequently confirmed in mice, using a NOTCH1 blocking antibody [160].

The growth of new vascular network in the primary tumor, also called neo-angiogenesis, is also critical for local invasion, i.e., the migration of cancer cells into the surrounding tissues. Notch signaling is highly active in endothelial cells and blood and lymph vessels. The presence of DLL4 and JAG1 ligands on endothelial cells is known to regulate the process of angiogenesis (Figure 5).

Expression of DLL4 is increased through VEGF/VEGFR signaling [161], and the JAG1/NOTCH interaction reinforces expression of the mesenchymal-specific intermediate filament protein vimentin [126]. In vitro and in vivo cancer models have shown that active Notch regulates the expression of adhesion molecule VCAM1 ensuring cancer cell migration across capillaries and blood vessels [162]. Additional studies showed that amino-terminal enhancer of split (AES), a suppressor of the transcriptional activity of Notch, leads to a reduced potential for invasion in colon and prostate cancers. Deletion of *Aes* in a mouse model of colon cancer resulted in marked elevation of Notch activity and the increase in the cells’ ability to intravasate [163,164]. It is important to keep in mind that vasculature is Notch positive under physiological conditions.

## 6. Notch Signaling and Metastasis

Metastasis is the spread of neoplastic cells from the primary site of the tumor to distant locations. Metastasizing tumor cells need to penetrate first into adjacent tissues and later colonize distant places, reached via the bloodstream and the lymphatic system. Many studies report that triggering the EMT process may self-regulate itself, but the precise mechanisms remain incompletely understood. Is EMT required for metastasis? We have seen that the interplay between EMT and Notch signaling could contribute to vascular intravasation [133,165,166]. However, it remains unclear which exact role Notch signaling may play in the context of distant metastasis. Latest data have identified an intermediate state between the epithelial and mesenchymal phenotypes, known as hybrid EMT (hyb-E/M), that is characterized by maintaining simultaneous epithelial (such as cell–cell adhesion) and mesenchymal (migration) features. Such transition states may correspond to the “tumor cell plasticity” observed in many cancer cell lines, which enables epithelial tumor cells to rapidly adjust to changes in their environment and also promotes invasion. The hyb-E/M state plays crucial roles in metastasis of cell clusters via bloodstream [167,168]. Recent data show that hyperactive Notch is not simply inducing EMT–it is more likely to fine-tune EMT. It appears that a co-ordinated interplay between NOTCH and EMT supports local (neo-)angiogenesis and lymph-angiogenesis (Figure 3), and ultimately promotes local metastasis and malignant cell proliferation (Figure 5). The mechanisms behind distant metastasis are less well understood than those related to tumor cell invasion and local migration. Mounting evidence suggests that cells may not even actively engage in an overt EMT status when they metastasize. In fact, deletion of key drivers of EMT like Snail, Slug, and Twist are dispensable for metastasis in a mouse model of pancreatic cancer [169]. In addition, several other EMT marker genes were not expressed during the metastatic process, e.g., of spontaneous lung cancer in a mouse model [36]. After intravasation of blood vessels, cancer cells may enter the blood stream either as single circulating tumor cells (CTC) or as clusters of CTCs, often together with endothelial and stromal cells (Figure 5). The activity of the Notch signaling pathway is important for the formation of such CTC clusters [170,171,172]. However, the mechanisms by which Notch signaling supports the metastatic potential of such heterogeneous clusters is an unresolved topic. The studies of Boareto et al. show that specific Notch–Jagged interactions promote cancer cells to undergo the previously mentioned hyb-E/M phenotype. This status may promote the formation of such cell clusters and stabilize them in the blood stream, thus also favoring extravasation [139] (Figure 5). These cells simultaneously express E-cadherin and vimentin as well as other epithelial and mesenchymal markers and have been found capable of metastasizing. These studies are of strong clinical relevance and may directly relate to the connection between invasive cancer cells and distant metastasis. Furthermore, the infiltration of neutrophils due to TGFβ stimuli is a critical factor for NOTCH1-induced metastasis in CRC [64]. Neutrophils have also been found to escort and thus protect circulating tumor cells within the blood stream. This may later also support extravasation and the formation of distant metastases [173] that successfully proliferate. Several other, often rather isolated and poorly connected, findings also point towards a potential role of Notch genes and Notch signaling in metastasis. For example, mice lacking Dll4 in endothelial cells form smaller (Lewis lung carcinoma) tumors with reduced expression of EMT markers (Snail1, Twist, Slug and Tgf-b) and stemness markers. This is associated with a reduced capacity for metastatic potential [166]. Dll4 also plays a key role in regulating angiogenesis, which will be discussed in detail below.

In this context, it is revealing that single, isolated CSCs have never been shown to successfully metastasize to other organs. This may be due to the high likelihood for single CSCs to perish due to anoikis, which is a form of attachment-depleted apoptosis. In contrast, it appears to be clusters of CSCs that are capable to form distant lesions in animal models [174]; possibly due to the supporting functions of Notch signaling within such heterogeneous aggregates. Interestingly, involvement of different Notch ligands may result in different CSC^-^ cluster sizes and phenotypes. Where JAG1^+^ clusters have a distinguished hyb-E/M phenotypes, DLL^+^ clusters have either epithelial or mesenchymal phenotypes [139,175]. The reason for such clusters to be successful in metastasis may relate to their advanced adhesion and collective migration capacity, promoted by co-expression of epithelial and mesenchymal genes like E-cadherin and Ovo Like Zinc Finger 2 (OVOL2; epithelial), as well as MMPs, vimentin, ZEB, Snail, and Twist (mesenchymal markers) [139]. This highly dynamic, hybrid phenotype not only renders these CTC cell clusters capable of surviving, in the blood circulation, but may also support extravasation by degradation of the basement membranes at the distant sites via MMPs. In addition, a good degree of stemness may also protect such migrating cells in the new environment from anoikis and help to evade the immune system at these sites. Circulating BrCa cell clusters that are positive to Jag^+^, CD24^Hi^ (epithelial marker), and CD44^Hi^ (mesenchymal and stemness cell marker) are particularly capable, able to repopulate distant localizations and successfully metastasise [139]. The combination of markers CD24^Hi^/CD44^Hi^ is also a characteristic hyb-E/M signature. Populations of these hyb-E/M cells have also been shown to be enriched after chemotherapeutic treatment, suggesting that these are also typical characteristics of chemo-resistant cells. The pro-invasive and pro-metastatic hyb-E/M phenotype is likely to be maintained via Notch signaling, which also prevents the cells from entering full-blown EMT status. This has been determined via mathematical means by Bocci and collaborators [143] and later also experimentally proven. In these studies, Notch modulation (or moderation) mainly occurred via the repressive functions of NUMB protein, an inhibitor of Notch intracellular signaling. This fine-tuned mitigation of Notch signaling may be sufficient for the cells to block entering full EMT. A comparable metered regulation of Notch signaling by Numb (Numb^Low^ and Notch^Hi^) has also been shown in TNBC lines [143]. Clinical outcome data from numerous lung, breast, and ovarian studies show a clear, significant correlation between NUMB expression and poor survival [143]. The mounting data on CSC clusters and hyb-E/M belongs, to date, to the most convincing and detailed molecular mechanisms of action of metastasis in several tumor types. We expect that much more data on this subject will be presented soon, and with it new insights into the roles of Notch signaling in this understudied phenomenon.

## 7. Notch Signaling in (Neo-)Angiogenesis

A critical aspect of successful metastasis, and the survival of tumor cells at sites distant from the primary tumor (to which they had been perfectly adapted), is vasculogenesis or neo-angiogenesis. Since Notch signaling is genuinely involved in vascular formation (angiogenesis during foetal and embryonal development), and expressed in blood vessels, capillaries, and endothelial cells, it is not surprising to find high levels of Notch signaling activity also in the newly formed vasculature around and within metastases, including both local lymph node and distant metastases.

In patients with HNSCC, one of the decisive parameters influencing the prognosis is the condition of the lymph nodes [176]. Hypoxia within the tumor tissues has been recently reported as the main cause for the shedding of larger numbers of CTC clusters, resulting in increasingly successful distant metastases [177]. This is in contrast to other studies showing that angiogenesis increases the metastatic process. For example, inhibiting or deleting DLL or Jag ligands reduces neo-angiogenesis in tumors, and resulted in more hypoxic tumors. These tumors only grow to smaller lesions, which is correlated with the formation of fewer metastatic lesions [5]. Additionally, data show that tumors with a high angiogenesis score were associated with recurrent metastasis, especially to the brain and bone [178]. Additionally, DLL4 expression is increased in distant metastases compared to primary HNSCC tumors and associated with poor outcome [56]. In HNSCC with overexpression of Jag1, xenografted together with human endothelial cells into mice, stimulated angiogenesis, and tumor invasion phenotypes [179].

Both Notch receptors and ligands are expressed in vessels, capillaries, and endothelial cells, and angiogenesis is promoted as tumors grow and become hypoxic. More specifically, co-activation of Notch signaling between endothelial cells and different cancer cells is promoted during neovascular formation. For example, it has been reported that blocking the action of Notch ligands such as DLL4 by a recombinant antibody can block angiogenesis and tumor progression–what was attributed to inactivation of hyperactive Notch signaling in BrCa [180]. Yet, it is more likely that this is due to blocking endothelial cells and neo-angiogenesis, which indirectly but nevertheless potently affects tumor growth, again demonstrating the relevance of the TME for tumor progression and therapy. Immunohistochemistry analyses for DLL4/JAG1 expression and microvascular formation of glioblastoma tumor tissue show enhanced expression of both Notch ligands in tumor vasculature and demonstrate a clear correlation with poor outcome in glioblastoma. Thus, DLL4 and JAG1 may have an inverse effect on tumor angiogenesis in glioblastoma [181]. This is further supported by another study demonstrating that mouse DLL4 or JAG1 are expressed in glioblastoma cells and reduced tumor cell proliferation in vitro, but stimulated tumor growth in vivo by modulating angiogenesis [182]. As results reported in breast cancer, tumors with a high score of angiogenesis also have enriched signaling pathways, including EMT, Notch, Hedgehog, and WNT/β-catenin signaling (162). Since metastasis is significantly reduced after knock-out of *DLL4*, this could be interpreted as mainly due to an inhibition of EMT (and thus Notch-linked), or alternatively due to a reduction of the number of circulating tumor cells [166]. A third option to explain the effects of Dll4 knock-down may relate to the inhibition of neo-angiogenesis and thus reduced access to the vasculature by cancer cells. The protective function of co-clustered neutrophils, which we have seen to associate with CSCs, may also be critical for the initiation of growth and neoangiogenesis right after extravasation [173].

## 8. Conclusions and Future Perspectives

In summary, we have mainly focused on the roles of Notch signaling in dynamic processes, such as EMT and cancer cell motility/invasion. We have shown that Notch receptors, ligands, and downstream genes are critical for those dynamic processes, that likely also promote cancer progression, such as vascular invasion and lymph-node and distant metastases. Nevertheless, the conclusions, especially on the putative connections between the Notch pathway and metastasis, cannot be finalized before much more research has elucidated the underlying mechanisms. This, however, will be difficult, as this type of research needs to take the complexity of Notch signaling and its dependency on the context in which it occurs into account. This also remains difficult by itself, as suitable model systems to investigate this connectivity of Notch with other pathways, cells, the ECM, and TME, and probably the immune system, are still rare. Similarly, more complex models systems, either in vitro or animal models (such as genetically engineered mouse models), may be required to firmly establish the true functionality of Notch signaling and Notch downstream genes and targets in cancer stem cell biology, the definition of the stem cell niche(s), and the resulting cancer dormancy. Last but not least, these aspects all contribute to the truly critical aspect, the question if we will be able to predict the response of patients to chemotherapy, by the means of (future) personalized medicine. The complexity of Notch signaling may be an outstanding hallmark directly related to individualized responses to cancer chemotherapy, even if Notch itself is not always directly targeted. Improved understanding of the interactions of the Notch pathway with other signaling pathways is likely to be a critical component of future individualized therapy and clinical decision-making. This accounts as much to Notch activity, its mutations, amplifications, and deletions observed in primary cancers, as to its incredibly flexible activity in the bigger context of living cancer tissues.

## Data Availability

Publicly available datasets were analyzed in this study. This data can be found here: https://www.cbioportal.org/study?id=hnsc_tcga (head and neck cancers), https://www.cbioportal.org/study?id=brca_tcga (breast cancers) and https://www.cbioportal.org/study?id=ccle_broad_2019 (cancer cell lines).
